# Systemic release of osteoprotegerin during oxaliplatin-containing induction chemotherapy and favorable systemic outcome of sequential radiotherapy in rectal cancer

**DOI:** 10.18632/oncotarget.8995

**Published:** 2016-04-26

**Authors:** Sebastian Meltzer, Erta Kalanxhi, Helga Helseth Hektoen, Svein Dueland, Kjersti Flatmark, Kathrine Røe Redalen, Anne Hansen Ree

**Affiliations:** ^1^ Department of Oncology, Akershus University Hospital, Lørenskog, Norway; ^2^ Institute of Clinical Molecular Biology, Akershus University Hospital, Lørenskog, Norway; ^3^ Institute of Clinical Medicine, University of Oslo, Oslo, Norway; ^4^ Department of Oncology, Oslo University Hospital, Norwegian Radium Hospital, Oslo, Norway; ^5^ Department of Tumor Biology, Oslo University Hospital, Norwegian Radium Hospital, Oslo, Norway; ^6^ Department of Gastroenterological Surgery, Oslo University Hospital, Norwegian Radium Hospital, Oslo, Norway

**Keywords:** osteoprotegerin, oxaliplatin, radiotherapy, metastasis, rectal cancer

## Abstract

In colorectal cancer, immune effectors may be determinative for disease outcome. Following curatively intended combined-modality therapy in locally advanced rectal cancer metastatic disease still remains a dominant cause of failure. Here, we investigated whether circulating immune factors might correlate with outcome. An antibody array was applied to assay changes of approximately 500 proteins in serial serum samples collected from patients during oxaliplatin-containing induction chemotherapy and sequential chemoradiotherapy before final pelvic surgery. Array data was analyzed by the Significance Analysis of Microarrays software and indicated significant alterations in serum osteoprotegerin (TNFRSF11B) during the treatment course, which were confirmed by osteoprotegerin measures using a single-parameter immunoassay. Patients experiencing increase in circulating osteoprotegerin during the chemotherapy had significantly better 5-year progression-free survival than those without increase (78% *versus* 48%; *P* = 0.009 by log-rank test). Hence, systemic release of this soluble tumor necrosis factor decoy receptor following the induction phase of neoadjuvant therapy was associated with favorable long-term outcome in patients given curatively intended chemoradiotherapy and surgery but with metastatic disease as the main adverse event. This finding suggests that osteoprotegerin may mediate or reflect systemic anti-tumor immunity invoked by combined-modality therapy in locally advanced rectal cancer.

## INTRODUCTION

In colorectal cancer, the influence of the tumor microenvironment with its immune effectors for disease outcome is increasingly acknowledged [1]. The recent study demonstrating favorable survival following immune checkpoint blockade in metastatic disease from mismatch repair-deficient tumors with a high density of immunogenic neo-antigens will obviously be regarded as a landmark contribution to the concept of immune modulation in colorectal cancer [2].

As a result of systematic improvements that include multimodal therapy, primarily neoadjuvant chemoradiotherapy (CRT) followed by surgery, long-term local control is commonly achieved in locally advanced rectal cancer (LARC) [3]. The unrivalled efficacy of radiotherapy in treatment of local tumor manifestations is a reflection of a delivered radiation dose that is commonly at the limit of normal tissue tolerance, and the improved survival outcomes resulting from the increasingly complex multimodality programs are often at the price of significant toxicities [4]. In pelvic curative CRT, enteritis that clinically presents as severe diarrhea may be a major adverse effect [5, 6].

Moreover, following curatively intended combined-modality therapy in LARC, a substantial number of patients will proceed to metastatic disease as a result of distant organ establishment of tumor cells with clonogenic potential [7]. Keeping this in mind, intriguing preclinical and clinical findings have suggested that the inflammatory, pro-immunogenic response to radiation damage within the tumor microenvironment may exert systemic anti-tumor activity at manifestations outside the radiation target volume. This comprises the so-called abscopal effect caused by radiation-induced immunogenic tumor cell death with the resulting cross-priming, via presentation of tumor antigens by dendritic cells, of tumor-targeting T lymphocytes [8-13].

Hence, within the frame of a prospective study for LARC patients (mainly T3–4 cases) given an intensified neoadjuvant treatment schedule and with long-term follow-up to observe metastatic progression [14], we investigated whether circulating inflammatory factors may relate to treatment toxicity and survival outcome. Study patients received short-course induction neoadjuvant chemotherapy (NACT) followed by long-course CRT before final pelvic surgery [14]. We employed an antibody array technology to monitor approximately 500 circulating proteins in serial serum samples collected throughout the full neoadjuvant course as a real-time approach to tumor responses and the constitutional and acquired physiology of the patients. In the resulting data set, the soluble tumor necrosis factor (TNF) decoy receptor osteoprotegerin (OPG) presented the most marked response to the neoadjuvant therapy (Table 1).

**Table 1:** Significantly altered serum proteins during neoadjuvant therapy

Post-NACT		Post-CRT				Evaluation			
ADIPOQ	1.15	ACVR1	1.10	IGFBP3	1.21	ADIPOQ	1.10	GRN	1.12
ANG	1.21	ADIPOQ	1.10	IGFBP7	1.12	ANG	1.11	IGF2	1.21
IGFBP2	1.15	ANG	1.21	IL1RAPL2	1.10	ANGPT2	1.16	IGFBP7	1.15
IL6ST	1.12	BMPR1A	1.11	IL27	1.12	BDNF	1.11	IL1RAPL2	1.11
NCAM1	1.12	CCL1	1.11	IL6ST	1.17	BMPR1A	1.18	IL22	1.12
SAA1	1.15	CCL11	1.12	LBP	1.16	CCL11	1.14	IL6ST	1.12
*TNFRSF11B*	*1.34*	CCL22	1.13	LEPR	1.11	CCR6	1.14	LIFR	1.12
		CCR6	1.10	PLAU	1.12	CD14	1.13	MMP2	1.10
		CD14	1.14	RARRES2	1.12	CSF1	1.19	NGFB	1.12
		CSF1	1.22	RELT	1.11	CTF1	1.12	NTF4	1.13
		EGFR	1.11	SAA1	1.27	CXCR1	1.19	RARRES2	1.23
		ERBB2	1.20	SIGLEC5	1.14	CXCR5	1.11	SIGLEC9	1.12
		FLT3LG	1.11	SIGLEC9	1.13	CXCR6	1.12	SLC2A2	1.19
		GCG	1.13	SLC2A2	1.13	ERBB2	1.11	THBS4	1.12
		GRN	1.16	TGFBR1	1.11	ERBB4	1.11	*TNFRSF11B*	*1.16*
		IGF2	1.18	THBS4	1.10	FLT3LG	1.12		
		IGFBP2	1.15	*TNFRSF11B*	*1.65*				
LCN2	0.65	CHRDL2	0.88	PDGFA	0.86	CHRDL2	0.86	PDGFA	0.88
LTBP1	0.87	CXCL2	0.88	PDGFB	0.89	FGF13	0.84	S100A12	0.84
MMP9	0.63	FGF13	0.83	PF4	0.85	LCN2	0.84	TMEFF2	0.89
		LCN2	0.73	PPBP	0.83	MMP9	0.74		
		LTBP1	0.80	S100A12	0.85				
		MMP9	0.68	THBS1	0.84				

Array values of the proteins were transformed to natural logarithms. The fold-change increase or decrease following induction neoadjuvant chemotherapy (post-NACT) and sequential chemoradiotherapy (post-CRT) and at evaluation of the neoadjuvant treatment, relative to baseline, is indicated on the right of each protein. Proteins are listed by their gene names (TNFRSF11B, in italic, corresponds to osteoprotegerin).

OPG (TNFRSF11B) is a glycoprotein expressed by several cell types, including dendritic cells and CD4-positive T lymphocytes, which binds the ligand of the receptor activator of nuclear factor-kappaB (RANK), RANKL [15-17]. The OPG/RANKL/RANK system is involved in a wide variety of biological processes and is essential for bone-resorbing osteoclast activity in bone remodeling [17-19]. In particular, RANKL-induced signaling is implicated in the antigen-specific interaction between dendritic cells and T lymphocytes, allowing the immune system to recognize and destroy abnormal cells with non-self antigens [19].

In this LARC study, systemic immunological markers and particularly the alterations in circulating levels of OPG during the neoadjuvant treatment course were correlated to progression-free survival (PFS) and treatment toxicity as prospectively assessed by Common Terminology Criteria for Adverse Events (CTCAE) scoring, a comprehensive grading system for adverse treatment effects [20]. This approach may provide insight into systemic anti-tumor immunity invoked by combined-modality radiotherapy in a patient population treated with curative intent but with a significant risk of metastatic disease as an adverse outcome.

## RESULTS

### Circulating proteins during the course of neoadjuvant therapy

The high-density antibody array, covering approximately 500 different proteins that include cytokines, growth factors, and proteinases among others, was applied to analyze serial serum samples collected from 66 of the study patients at baseline, at completion of four weeks of induction NACT (post-NACT) and the sequential 5-week CRT course (post-CRT), and at evaluation of the neoadjuvant treatment four weeks later. Analysis of the array data set by Significance Analysis of Microarrays (SAM) revealed significant changes in levels of a number of serum proteins during the neoadjuvant treatment course (Table 1) with OPG showing the strongest increase (mean fold-change from baseline of data transformed to natural logarithms) of 1.34 and 1.65, respectively, at the post-NACT and post-CRT sampling points.

### Circulating OPG and markers of systemic inflammation

**Figure 1:**
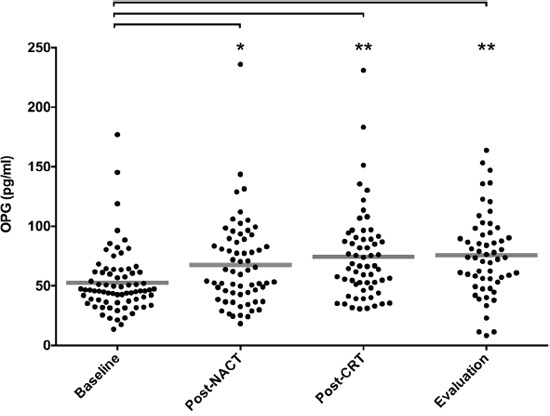
Serum OPG levels during neoadjuvant therapy Using the single-parameter immunoassay, OPG was measured in serum sampled from patients at baseline (*n* = 74), post-NACT (*n* = 64), post-CRT (*n* = 61), and at evaluation of the neoadjuvant treatment (*n* = 57). For each sample group, where mean value is indicated by a line, distribution of values was different from baseline (* *P* < 0.01, ** *P* < 0.0001; calculated by unpaired one-way analysis of variance).

Serum OPG was therefore assessed by a single-parameter immunoassay in samples collected at baseline, post-NACT, post-CRT, and evaluation. Increase in circulating OPG from the mean baseline value of 52.5 pg/ml to the mean value of 75.7 pg/ml at evaluation was observed (Figure 1). All available samples were entered into this analysis but with variability in the number of cases throughout the various sampling points (*n* = 57–74). One reason was that several patients with CTCAE grade 3 diarrhea or other high-grade adverse treatment events were lost for further serum sampling when they were admitted at local hospitals. For other study patients, remaining serum lots could not be retrieved.

As seen from Table 2, the baseline OPG measures (range 13.6–177 pg/ml; *n* = 74) demonstrated significant correlations with patients' age as well as common markers of systemic inflammation, such as baseline erythrocyte sedimentation rate (ESR) and post-NACT neutrophil-to-lymphocyte ratio (NLR). Related to this, negative correlation with the serum albumin level and hence, positive correlation with the level of ionized calcium were seen. Moreover, at each sampling point during active therapy, correlation was found between the level of OPG and the actual monocyte count (Supplementary Figure 1). Correlation between age and serum OPG has also been reported by other investigators [21, 22], and further analyses were therefore conducted on age-adjusted OPG values.

**Table 2:** Correlations between baseline serum osteoprotegerin levels and other patient factors

	*n* (%)	Correlation	*P*-value
Age (years)	74 (100)	0.30	0.009
Body mass index (kg/m^2^)	73 (99)	−0.05	0.649
Hemoglobin (g/dl)	74 (100)	−0.14	0.244
Thrombocytes (10^9^/l)	74 (100)	0.14	0.252
Leukocytes (10^9^/l)	74 (100)	0.25	0.035
Neutrophils (10^9^/l)	74 (100)	0.19	0.097
Lymphocytes (10^9^/l)	72 (97)	0.16	0.173
Monocytes (10^9^/l)	71 (96)	0.34	0.004
NLR Baseline	72 (97)	0.03	0.800
NLR Post-NACT	62 (84)	0.33	0.009
Albumin (g/l)	74 (100)	−0.25	0.032
Ionized calcium (mmol/l)	72 (97)	0.24	0.042
ESR (mm/h)	67 (91)	0.26	0.033
Carcinoembryonic antigen (μg/l)	74 (100)	0.12	0.315

Determined by Pearson product correlation.

Abbreviations: ESR, erythrocyte sedimentation rate; NACT, neoadjuvant chemotherapy; NLR, neutrophil-to-lymphocyte ratio.

### Patient parameters and disease outcome

When last censored, median follow-up time for the whole study population of 85 cases within the current report was 59 months (range 3–66). Three patients had experienced local recurrence as the first event of disease relapse. In addition, 28 patients had metastatic progression as the first event, with liver as the dominantly affected organ (16 cases) followed by lungs (eight cases) and other sites (four cases). Hence, PFS was chosen as the relevant long-term endpoint. As seen in Table 3, univariate Cox regression analysis revealed significant association, reflected by hazard ratio (HR), between a PFS event and poor histologic (yp) tumor-node (TN) down-staging (ypT3–4: HR 4.05; ypN1–2: HR 3.83) and poor tumor regression grade (TRG) score (TRG 3–5: HR 2.61) in the surgical specimens from mainly T3–4 cases. Moreover, favorable PFS was associated with older age (HR 0.14), higher baseline hemoglobin (HR 0.04), less treatment toxicity (CTCAE grade 0–2 diarrhea: HR 0.37), and interestingly, dose reduction of oxaliplatin during CRT (HR 0.48) and a longer time span from CRT completion to surgery (HR 0.02). In contrast, both high baseline ESR (HR 1.85) and post-NACT NLR (HR 1.89) were associated with adverse PFS. And with reference to the correlations between baseline serum OPG measures and these inflammation markers (Table 2), high OPG levels (age-adjusted) were also associated with unfavorable PFS (HR 3.33). In multivariate analysis, entering baseline variables with significant association with PFS, low hemoglobin remained associated with adverse PFS while OPG (HR 2.55; *P* = 0.051) failed to reach significance (Supplementary Table 1).

**Table 3:** Progression-free survival – univariate analysis

		*n* (%)	Median (range)	HR (95% CI)	*P*-value
Sex	Female	35 (41)			
	Male	50 (59)		0.98 (0.48–2.00)	0.957
TN stage	T2	5 (6)			
	T3	50 (59)			
	T4	30 (35)		1.71 (0.91–3.23)	0.095
	N0	10 (12)			
	N1	9 (11)			
	N2	65(77)		1.20 (0.68–2.10)	0.536
	ND	1			
ypTN stage^a^	ypT0–2	43 (51)			
	ypT3–4	41 (49)		4.05 (1.80–9.13)	0.001
	ND	1			
	ypN0	57 (67)			
	ypN1–2	27 (33)		3.83 (1.86–7.91)	0.000
	ND	1			
TRG score^a^	TRG 1–2	60 (70)			
	TRG 3–5	24 (30)		2.61 (1.26–5.38)	0.010
	ND	1			
CTCAE score^b^	CTCAE 0–2	52 (61)		0.37 (0.16–0.87)	0.023
	CTCAE 3	12 (14)			
	ND	21 (25)			
NACT	Full dose	77 (90)			
	Reduction	8 (10)		0.59 (0.14–2.48)	0.471
Oxaliplatin during CRT	Full dose	20 (24)			
	Reduction	65 (76)		0.48 (0.23–0.99)	0.048
Capecitabine during CRT	Full dose	28 (33)			
	Reduction	57 (67)		0.87 (0.42–1.81)	0.706
Radiation	Full dose	80 (94)			
	Reduction	5 (6)		0.53 (0.07–3.90)	0.530
Age (years)		85 (100)	59 (30–73)	0.14 (0.03–0.71)	0.018
Body mass index (kg/m^2^)		84 (99)	24.6 (17.9–34.8)	2.50 (0.17–37.5)	0.509
Time from CRT completion to surgery (weeks)		66 (78)	7.4 (4.4–10.4)	0.02 (0.00–0.41)	0.010
Hemoglobin (g/dl)		85 (100)	13.9 (9.3–16.3)	0.04 (0.00–0.51)	0.013
Thrombocytes (10^9^/l)		85 (100)	320 (182–990)	2.90 (0.89–9.43)	0.078
Leukocytes (10^9^/l)		85 (100)	7.3 (4.0–16.2)	1.26 (0.34–4.65)	0.726
Neutrophils (10^9^/l)		85 (100)	4.8 (2.3–12.8)	1.27 (0.47–3.44)	0.638
Lymphocytes (10^9^/l)		83 (98)	1.8 (0.90–3.9)	0.74 (0.22–2.50)	0.622
Monocytes (10^9^/l)		82 (96)	0.6 (0.2–1.4)	0.81 (0.31–2.11)	0.662
Baseline NLR		83 (98)	2.4 (1.2–9.3)	1.28 (0.56–2.91)	0.561
Post-NACT NLR		70 (82)	1.4 (0.6–15.8)	1.89 (1.08–3.31)	0.025
Albumin (g/l)		85 (100)	42 (25–48)	0.32 (0.01–17.4)	0.574
Ionized calcium (mmol/l)		83 (98)	2.37 (2.22–2.76)	0.59 (0.00–11959)	0.916
ESR (mm/h)		76 (89)	14 (2–106)	1.85 (1.10–3.11)	0.021
Carcinoembryonic antigen (μg/l)		85 (100)	3 (0–122)	1.02 (0.90–1.16)	0.715
Baseline OPG (pg/ml/year)		74 (87)	0.9 (0.2–2.6)	3.33 (1.24–8.94)	0.017
Post-NACT OPG (pg/ml/year)		64 (75)	1.2 (0.3–4.6)	1.42 (0.61–3.29)	0.419
Post-CRT OPG (pg/ml/year)		61 (72)	1.2 (0.4–4.0)	1.56 (0.63–3.91)	0.339
Evaluation OPG (pg/ml/year)		57 (67)	1.3 (0.1–3.0)	0.80 (0.37–1.69)	0.552

a One patient had disease progression in the pelvic cavity during neoadjuvant treatment and therefore proceeded to palliative surgery. As consequence, histologic tumor response data was missing, and the single case was omitted from these analyses.

b Twenty-one patients had either baseline Common Terminology Criteria for Adverse Events (CTCAE) diarrhea scores >0 or did not report maximum CTCAE grade diarrhea, and were therefore omitted from this analysis.

Other abbreviations: CI, confidence interval; CRT, chemoradiotherapy; ESR, erythrocyte sedimentation rate; HR, hazard ratio; NACT, neoadjuvant chemotherapy; ND, not determined; NLR, neutrophil-to-lymphocyte ratio; OPG, osteoprotegerin; TN, tumor-node; TRG, tumor regression grade; ypTN, histologic TN stage.

### Changes in circulating OPG during neoadjuvant therapy

**Figure 2:**
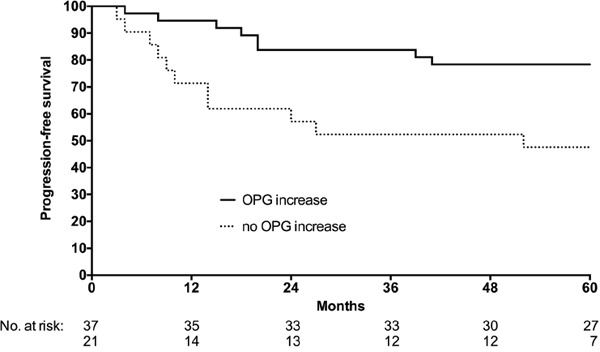
Serum OPG levels and PFS Patients were separated into cases with (solid line) or without (dashed line) increase in circulating OPG levels from baseline to completion of induction neoadjuvant chemotherapy, as measured by the single-parameter immunoassay. The difference between the two groups was significant (log-rank test; *P* = 0.009).

With reference to the observed population-based increase in circulating OPG throughout the course of neoadjuvant combined-modality therapy (Figure 1), we investigated whether alteration in the individual patient's serum OPG level at each of the sampling points (relative to the previous one) might reflect the contribution of the respective completed therapy component to overall outcome and tolerance. Firstly, for PFS, only the change at NACT completion (relative to baseline) was predictive with estimated 5-year PFS rate of 78% *versus* 48% when separating available cases (*n* = 58 for this particular analysis) with increase in the serum OPG level during NACT from those without (Figure 2), even though patients with and without increase were equally distributed with regard to ypTN status and TRG scores (Supplementary Table 2).

Secondly, in this study population, pelvic CRT was the treatment modality that caused intestinal toxicity [14]. Since OPG does not show tumor-specific expression, its release into the circulation might, in susceptible individuals, reflect treatment-induced enteritis that clinically presents as diarrhea. To precisely define adverse events specifically associated with the therapy, patients reporting diarrhea at baseline (as presenting symptom of their disease) or with the maximum CTCAE score missing were omitted from this analysis. The remaining cases were categorized according to the maximum CTCAE score recorded during the neoadjuvant treatment. No patient reported higher than CTCAE grade 3 diarrhea. Hence, categories consisted of cases devoid of diarrhea (CTCAE grade 0) throughout the treatment course and cases reporting maximum CTCAE grade 1, 2, and 3 diarrhea, respectively. On comparison of the CTCAE grade 3 category (reflecting injury of grave severity that will trigger treatment adjustment and usually involves hospital admission) with CTCAE grades 0–2 (corresponding to no higher than moderate toxicity) grouped together, the change in serum OPG level during NACT was not found to be associated with adverse CTCAE grade diarrhea (Supplementary Table 2).

Of note, this specific analysis was compromised by the low number of serum samples from CTCAE grade 3 cases. Moreover, the neoadjuvant regimen was adjusted according to toxicity by reducing doses of or entirely discontinuing oxaliplatin, capecitabine, or radiotherapy in that order of priority, in accordance with the relative importance of the three therapeutic components. As a consequence (Table 3), CTCAE grade 3 diarrhea was reported only by 19% of all cases (with known maximum score) within the current report, and 76% and 67% had dose reduction of oxaliplatin and capecitabine, respectively. In contrast, 94% of patients received the total prescribed radiation dose. Only five patients had a break during delivery (data not shown).

## DISCUSSION

In this rectal cancer cohort with locally advanced tumors, elevated levels of systemic inflammation markers on commencement of neoadjuvant combined-modality therapy were associated with adverse PFS. On the contrary, the association between increase in circulating OPG following induction NACT and favorable outcome in patients given curatively intended sequential CRT, but with metastatic disease as the main adverse event, suggests that systemic anti-tumor effects may have been invoked by the combined-modality therapy in patients who achieved long-term disease control. One might hypothesize that OPG mediates or reflects immune effector priming by the induction NACT.

The study population within the current report revealed significant associations between adverse outcome and well-known unfavorable clinical parameters, such as poor histologic TN down-staging and tumor regression following the neoadjuvant therapy. In a similar fashion to our study, recent analysis of large retrospective series has indicated that a prolonged interval between completion of long-course neoadjuvant therapy and surgery may increase the likelihood of achieving excellent histologic tumor response [23-25]. The observation that patients who did not receive the full protocol-specified oxaliplatin dose during CRT had favorable PFS is in line with reports (though from retrospective analyses), where neutropenia from oxaliplatin-containing chemotherapy was associated with improved survival in metastatic colorectal and gastric cancer [26, 27]. In this context, improved tumor response has been related to strong oxaliplatin-DNA adduct formation in white blood cells [28]. Additionally, in agreement with our findings, previous studies have reported low hemoglobin and circulating lymphocytes being adverse prognostic factors in LARC patients given neoadjuvant therapy [29, 30]. The association between CTCAE grade 3 diarrhea and unfavorable PFS in our study is also in accordance with the recognition that an adverse event causing interruption in the radiation delivery is likely to have a negative influence on the probability of tumor control [4]. With all of this taken together, the current study population should be regarded as representative for LARC, and it is tempting to postulate that our descriptive data may reflect biologically significant mechanisms in outcome to combined-modality therapy in rectal cancer.

Interestingly, the TNF decoy receptor OPG showed apparently contradictory biological behavior. On the one hand, high baseline serum OPG levels were associated with unfavorable PFS, though it just failed to reach significance in multivariate analysis. On the other hand, patients with a rise in serum OPG measures during the neoadjuvant treatment course had better PFS than those without.

On binding to RANK, RANKL-induced signaling results in osteoclast activation, which is central for bone homeostasis. In this regulatory loop, OPG (literally, protector of bone) acts as a soluble receptor that deprives RANK of its ligand [17, 18]. The correlations observed in the present study between high baseline OPG levels and serological markers that in clinical practice frequently are deranged in cancer patients with poor prognosis, including elevated ionizing calcium, may suggest that high *de novo* circulating OPG reflects a rescue response to the high osteoclast activity that is associated with disease of grave severity and not an adverse disease mechanism in itself. Alternatively, elevated serum OPG at baseline may mirror elevated OPG expression in primary CRC tumors with high propensity to metastasize, which has been explained by autocrine OPG-dependent prevention of tumor cell apoptosis via inhibitory binding of the TNF-related apoptosis-inducing ligand [31, 32].

Moreover, the level of circulating OPG increased throughout the neoadjuvant treatment course and correlated with the actual monocyte count at each sampling point. Since OPG is expressed by monocyte-derived dendritic cells [15], these observations suggest that the study treatment may have caused dendritic cell activation. When separating study patients into cases who did or did not experience a rise in serum OPG levels during NACT, significantly better PFS was found for the former group, even though patients with and without increase were equally distributed among cases with favorable and unfavorable histologic ypTN and TRG outcomes in the surgical specimens. This finding indicates that the serum OPG response may add independent prognostic and indeed, biological information to current routine practice.

Importantly, we could not exclude the possibility that alterations in serum content of OPG reflected deleterious normal tissue effects. With the particular study design, the pelvic CRT caused intestinal toxicity [14], and the normal bowel might therefore contribute to alterations in circulating levels of OPG. Already a decade ago, a preclinical study using a mouse model demonstrated that OPG-dependent quenching of the RANKL-RANK interaction inhibited the severity of T lymphocyte-mediated bowel inflammation by decreasing the number of local, colonic dendritic cells [33]. However, no correlations were found between NACT-induced alterations in serum OPG and diarrhea scores. Essentially, the study displayed low incidence of CTCAE grade 3 diarrhea, probably reflecting the precaution criteria in the study protocol. The safety concerns may have precluded the quest for circulating markers of treatment toxicity.

Finally, in this patient cohort, we recently observed that the short-course induction NACT in patients with favorable PFS caused a strong increase in circulating carbonic anhydrase IX [34]. This tumor-specific enzyme is induced by tumor hypoxia and causes microenvironmental acidification, which is recognized as a main mechanism in resistance to cytotoxic therapy and metastatic progression [35-37]. Our data was interpreted as NACT-specific eradication of hypoxic tumor components, resulting in enhancement of CRT efficacy [34]. Given the parallel increase in circulating OPG levels associated with favorable outcome, it is tempting to speculate that the induction NACT also caused immune effector priming of the tumor, resulting in enhanced systemic anti-tumor immunity from the sequential CRT. The integral biology of local tumor microenvironmental and systemic immune responses will probably position itself within clinical radiation oncology practice in the near future [38].

## MATERIALS AND METHODS

### Patients, treatment, and serum sampling for LARC-RRP

The study protocol (ClinicalTrials.gov NCT00278694) was approved by the Institutional Review Board and Regional Committee for Medical and Health Research Ethics and was in accordance with the Helsinki Declaration. Written informed consent was required for participation. The study population of 85 cases within the current report was enrolled from October 5, 2005 through March 3, 2010. Patient eligibility criteria, evaluation procedures, and review procedures of follow-up have been described previously [39]. The neoadjuvant treatment protocol consisted of two cycles of NACT (the Nordic FLOX regimen: oxaliplatin 85 mg/m^2^ on day 1 and bolus fluorouracil 500 mg/m^2^ and folinic acid 100 mg on days 1 and 2 every second week) followed by CRT. Radiation was delivered in 2-Gy fractions five days per week over a 5-week period, with concomitant weekly oxaliplatin 50 mg/m^2^ and capecitabine 825 mg/m^2^ twice daily on days of radiotherapy. Formal recording of adverse events according to CTCAE version 3.0 was performed at baseline, at completion of NACT (post-NACT) and CRT (post-CRT), and at the time of treatment evaluation four weeks after its completion. Surgery was planned 6–8 weeks after completion of the neoadjuvant treatment. In accordance with national guidelines, patients did not proceed to further therapy. Study serum samples were collected at baseline, post-NACT, post-CRT, and at the time of treatment evaluation, and were stored at −80°C until analysis. Routine blood tests were done within the standard patient follow-up.

### Antibody array technology and data analysis

Serum samples (baseline, *n* = 66; post-NACT, *n* = 61; post-CRT, *n* = 59; evaluation, *n* = 55) were analyzed with a high-density antibody array (AAH-BLG-1; RayBiotech, Inc., Norcross, GA, USA) at the Genomics Core Facility, Oslo University Hospital. Serum proteins were biotinylated and added onto glass slides pre-printed with 507 capture antibodies. Bound proteins (in duplicates) were detected with a streptavidin-conjugated fluorescent dye, HiLytePlus™ 555 (60672-Plus555; AnaSpec, Inc., Freemont, CA, USA), and the arrays were scanned for fluorescence using the Agilent scanner G2505C (Agilent Technologies, Santa Clara, CA, USA). GenePix version 6.0 (Molecular Devices Corporation, Union City, CA, USA) was used to convert array image spots to numerical values. The array data is available in the Gene Expression Omnibus repository by accession number GSE65622.

Following data processing as detailed in the Supplementary information within the deposited data, the data was transformed to natural logarithms, and changes in circulating proteins during the neoadjuvant treatment course were determined with the SAM software version 5.0 employing paired analysis and a false discovery rate cut-off of 10% [40]. Herein, each protein that within the study population was significantly altered during the neoadjuvant treatment received a score on the basis of its change relative to the standard deviation of repeat measurements. The software handles any missing data by imputation using the *K*-nearest neighbor method [40].

### Single-parameter analysis of OPG

Serum samples (baseline, *n* = 74; post-NACT, *n* = 64; post-CRT, *n* = 61; evaluation, *n* = 57) were analyzed for OPG levels using a single-parameter immunoassay (ELH-OPG; RayBiotech, Inc.), according to the instructions manual. Before analysis, serum samples were diluted 1:4. All samples were analyzed in duplicates.

### Study endpoints

The clinical endpoints were treatment toxicities (CTCAE scores) during neoadjuvant therapy, histologic tumor response, and PFS. Follow-up data was censored on August 8, 2013. The resected tumor specimens were histologically evaluated for treatment response according to standard staging (ypTN; TNM version 5). In this patient population of locally advanced tumors (mainly T3–4 cases), ypT0–2 results were considered as good response and correspondingly, ypT3–4 results were regarded as poor tumor shrinkage. Moreover, histologic tumor response was graded within one of five TRG categories, spanning from the absence of residual tumor cells in the resected specimen (pathologic complete response; TRG 1) to the lack of morphologic signs of tissue response to treatment (TRG 5) [41]. Of note, when responding to neoadjuvant treatment, LARC frequently shows fragmentation into microscopic residual disease [42]. Consequently, it is rational to group TRG 2 together with TRG 1 as good histologic regression and correspondingly, the range of TRG 3–5 scores as poor response.

### Statistical analysis

Analyses were preformed using IBM SPSS Statistics for Windows version 23.0 or GraphPad Prism version 6.0h. Correlations between continuous data were determined by Pearson product correlation analysis after transformation to natural logarithms for symmetric distribution. Continuous data was described with either mean and standard deviation or median and range, and groups were compared using one-way analysis of variance with Holm-Sidak's test for multiple comparisons. Categorical data was compared using Chi-square test or Fisher's exact test when small numbers were involved. Estimated 5-year PFS was calculated from the time of study enrolment to the date of recurrent disease (diagnosis of local recurrence or distant metastasis), death of any cause, or end of follow-up (five years after the date of surgery), whichever came first. Crude differences in survival were assessed by the log-rank test and visualized by the Kaplan-Meier method. Associations between selected variables and PFS were modeled with univariate and multivariate Cox regression analysis. The results were expressed as HR with 95% confidence interval. All tests were two-sided. *P*-values less than 0.05 were considered statistically significant.

## SUPPLEMENTARY FIGURES AND TABLES



## References

[1] Galon J, Mlecnik B, Bindea G, Angell HK, Berger A, Lagorce C, Lugli A, Zlobec I, Hartmann A, Bifulco C, Nagtegaal ID, Palmqvist R, Masucci GV (2014). Towards the introduction of the 'Immunoscore' in the classification of malignant tumours. J Pathol.

[2] Le DT, Uram JN, Wang H, Bartlett BR, Kemberling H, Eyring AD, Skora AD, Luber BS, Azad NS, Laheru D, Biedrzycki B, Donehower RC, Zaheer A (2015). PD-1 blockade in tumors with mismatch-repair deficiency. N Engl J Med.

[3] Smith JJ, Garcia-Aguilar J (2015). Advances and challenges in treatment of locally advanced rectal cancer. J Clin Oncol.

[4] Ree AH, Meltzer S, Flatmark K, Dueland S, Kalanxhi E (2014). Biomarkers of treatment toxicity in combined-modality cancer therapies with radiation and systemic drugs: study design, multiplex methods, molecular networks. Int J Mol Sci.

[5] Kavanagh BD, Pan CC, Dawson LA, Das SK, Li XA, Ten Haken RK, Miften M (2010). Radiation dose-volume effects in the stomach and small bowel. Int J Radiat Oncol Biol Phys.

[6] Jabbour SK, Patel S, Herman JM, Wild A, Nagda SN, Altoos T, Tunceroglu A, Azad N, Gearheart S, Moss RA, Poplin E, Levinson LL, Chandra RA (2012). Intensity-modulated radiation therapy for rectal carcinoma can reduce treatment breaks and emergency department visits. Int J Surg Oncol.

[7] Ree AH, Redalen KR (2015). Personalized radiotherapy: concepts, biomarkers and trial design. Br J Radiol.

[8] Formenti SC, Demaria S (2013). Combining radiotherapy and cancer immunotherapy: a paradigm shift. J Natl Cancer Inst.

[9] Golden EB, Frances D, Pellicciotta I, Demaria S, Helen Barcellos-Hoff M, Formenti SC (2014). Radiation fosters dose-dependent and chemotherapy-induced immunogenic cell death. Oncoimmunology.

[10] Golden EB, Formenti SC (2014). Is tumor (R)ejection by the immune system the “5th R” of radiobiology?. Oncoimmunology.

[11] Victor CT, Rech AJ, Maity A, Rengan R, Pauken KE, Stelekati E, Benci JL, Xu B, Dada H, Odorizzi PM, Herati RS, Mansfield KD, Patsch D (2015). Radiation and dual checkpoint blockade activate non-redundant immune mechanisms in cancer. Nature.

[12] Filatenkov A, Baker J, Mueller AM, Kenkel J, Ahn GO, Dutt S, Zhang N, Kohrt H, Jensen K, Dejbakhsh-Jones S, Shizuru JA, Negrin RN, Engleman EG (2015). Ablative tumor radiation can change the tumor immune cell microenvironment to induce durable complete remissions. Clin Cancer Res.

[13] Golden EB, Chhabra A, Chachoua A, Adams S, Donach M, Fenton-Kerimian M, Friedman K, Ponzo F, Babb JS, Goldberg J, Demaria S, Formenti SC (2015). Local radiotherapy and granulocyte-macrophage colony-stimulating factor to generate abscopal responses in patients with metastatic solid tumours: a proof-of-principle trial. Lancet Oncol.

[14] Dueland S, Ree AH, Grøholt KK, Saelen MG, Folkvord S, Hole KH, Seierstad T, Larsen SG, Giercksky KE, Wiig JN, Boye K, Flatmark K (2016). Oxaliplatin-containing preoperative therapy om locally advanced rectal cancer: local response, toxicity and long-term outcome. Clin Oncol (R Coll Radiol).

[15] Schoppet M, Henser S, Ruppert V, Stubig T, Al-Fakhri N, Maisch B, Hofbauer LC (2007). Osteoprotegerin expression in dendritic cells increases with maturation and is NF-kappaB-dependent. J Cell Biochem.

[16] Chakravarti A, Marceau AA, Flamand L, Poubelle PE (2008). Normal human primary CD4+ T lymphocytes synthesize and release functional osteoprotegerin in vitro. Lab Invest.

[17] Leibbrandt A, Penninger JM (2008). RANK/RANKL: regulators of immune responses and bone physiology. Ann NY Acad Sci.

[18] Lacey DL, Boyle WJ, Simonet WS, Kostenuik PJ, Dougall WC, Sullivan JK, Martin JS, Dansey R (2012). Bench to bedside: elucidation of the OPG–RANK–RANKL pathway and the development of denosumab. Nat Rev Drug Discov.

[19] Criscitiello C, Viale G, Gelao L, Esposito A, De Laurentiis M, De Placido S, Santangelo M, Goldhirsch A, Curigliano G (2015). Crosstalk between bone niche and immune system: osteoimmunology signaling as a potential target for cancer treatment. Cancer Treat Rev.

[20] Trotti A, Colevas AD, Setser A, Rusch V, Jaques D, Budach V, Langer C, Murphy B, Cumberlin R, Coleman CN, Rubin P (2003). CTCAE v3. 0: development of a comprehensive grading system for the adverse effects of cancer treatment. Semin Radiat Oncol.

[21] Kudlacek S, Schneider B, Woloszczuk W, Pietschmann P, Willvonseder R (2003). Serum levels of osteoprotegerin increase with age in a healthy adult population. Bone.

[22] Szulc P, Hofbauer LC, Heufelder AE, Roth S, Delmas PD (2001). Osteoprotegerin serum levels in men: correlation with age, estrogen, and testosterone status. J Clin Endocrinol Metab.

[23] Marijnen CA (2015). Organ preservation in rectal cancer: have all questions been answered?. Lancet Oncol.

[24] Wolthuis AM, Penninckx F, Haustermans K, De Hertogh G, Fieuws S, Van Cutsem E, D'Hoore A (2012). Impact of interval between neoadjuvant chemoradiotherapy and TME for locally advanced rectal cancer on pathologic response and oncologic outcome. Ann Surg Oncol.

[25] Sloothaak DA, Geijsen DE, van Leersum NJ, Punt CJ, Buskens CJ, Bemelman WA, Tanis PJ (2013). Optimal time interval between neoadjuvant chemoradiotherapy and surgery for rectal cancer. Br J Surg.

[26] Shitara K, Matsuo K, Takahari D, Yokota T, Inaba Y, Yamaura H, Sato Y, Najima M, Ura T, Muro K (2009). Neutropaenia as a prognostic factor in metastatic colorectal cancer patients undergoing chemotherapy with first-line FOLFOX. Eur J Cancer.

[27] Liu R, Huang M, Zhao X, Peng W, Sun S, Cao J, Ji D, Wang C, Guo W, Li J, Yin J, Zhu X (2015). Neutropenia predicts better prognosis in patients with metastatic gastric cancer on a combined epirubicin, oxaliplatin and 5-fluorouracil regimen. Oncotarget.

[28] Pieck AC, Drescher A, Wiesmann KG, Messerschmidt J, Weber G, Strumberg D, Hilger RA, Scheulen ME, Jaehde U (2008). Oxaliplatin-DNA adduct formation in white blood cells of cancer patients. Br J Cancer.

[29] Berardi R, Braconi C, Mantello G, Scartozzi M, Del Prete S, Luppi G, Martinelli R, Fumagalli M, Valeri G, Bearzi I, Marmovale C, Grillo-Ruggieri F, Cascinu S (2006). Anemia may influence the outcome of patients undergoing neo-adjuvant treatment of rectal cancer. Ann Oncol.

[30] Kitayama J, Yasuda K, Kawai K, Sunami E, Nagawa H (2011). Circulating lymphocyte is an important determinant of the effectiveness of preoperative radiotherapy in advanced rectal cancer. BMC Cancer.

[31] Tsukamoto S, Ishikawa T, Iida S, Ishiguro M, Mogushi K, Mizushima H, Uetake H, Tanaka H, Sugihara K (2011). Clinical significance of osteoprotegerin expression in human colorectal cancer. Clin Cancer Res.

[32] De Toni EN, Thieme SE, Herbst A, Behrens A, Stieber P, Jung A, Blum H, Goke B, Kolligs FT (2008). OPG is regulated by beta-catenin and mediates resistance to TRAIL-induced apoptosis in colon cancer. Clin Cancer Res.

[33] Ashcroft AJ, Cruickshank SM, Croucher PI, Perry MJ, Rollinson S, Lippitt JM, Child JA, Dunstan C, Felsburg PJ, Morgan GJ, Carding SR (2003). Colonic dendritic cells, intestinal inflammation, and T cell-mediated bone destruction are modulated by recombinant osteoprotegerin. Immunity.

[34] Hektoen HH, Flatmark K, Andersson Y, Dueland S, Redalen KR, Ree AH (2015). Early increase in circulating carbonic anhydrase IX during neoadjuvant treatment predicts favourable outcome in locally advanced rectal cancer. BMC Cancer.

[35] Begg AC, Stewart FA, Vens C (2011). Strategies to improve radiotherapy with targeted drugs. Nat Rev Cancer.

[36] Toustrup K, Sorensen BS, Nordsmark M, Busk M, Wiuf C, Alsner J, Overgaard J (2011). Development of a hypoxia gene expression classifier with predictive impact for hypoxic modification of radiotherapy in head and neck cancer. Cancer Res.

[37] Lu X, Kang Y (2010). Hypoxia and hypoxia-inducible factors: master regulators of metastasis. Clin Cancer Res.

[38] Barker HE, Paget JT, Khan AA, Harrington KJ (2015). The tumour microenvironment after radiotherapy: mechanisms of resistance and recurrence. Nat Rev Cancer.

[39] Folkvord S, Flatmark K, Dueland S, de Wijn R, Groholt KK, Hole KH, Nesland JM, Ruijtenbeek R, Boender PJ, Johansen M, Giercksky KE, Ree AH (2010). Prediction of response to preoperative chemoradiotherapy in rectal cancer by multiplex kinase activity profiling. Int J Radiat Oncol Biol Phys.

[40] Tusher VG, Tibshirani R, Chu G (2001). Significance analysis of microarrays applied to the ionizing radiation response. Proc Natl Acad Sci USA.

[41] Bouzourene H, Bosman FT, Seelentag W, Matter M, Coucke P (2002). Importance of tumor regression assessment in predicting the outcome in patients with locally advanced rectal carcinoma who are treated with preoperative radiotherapy. Cancer.

[42] Hole KH, Larsen SG, Groholt KK, Giercksky KE, Ree AH (2013). Magnetic resonance-guided histopathology for improved accuracy of tumor response evaluation of neoadjuvant treatment in organ-infiltrating rectal cancer. Radiother Oncol.

