# Supplementary material for: Systemic release of osteoprotegerin during oxaliplatin-containing induction chemotherapy and favorable systemic outcome of sequential radiotherapy in rectal cancer
Source: Oncotarget. 2016 Apr 26;7(23):34907–17. doi: 10.18632/oncotarget.8995 (PMC5085198; doi:10.18632/oncotarget.8995)
Supplement: Supplementary file 1 [file oncotarget-07-34907-s001.pdf]

SUPPLEMENTARY FIGURE AND TABLES

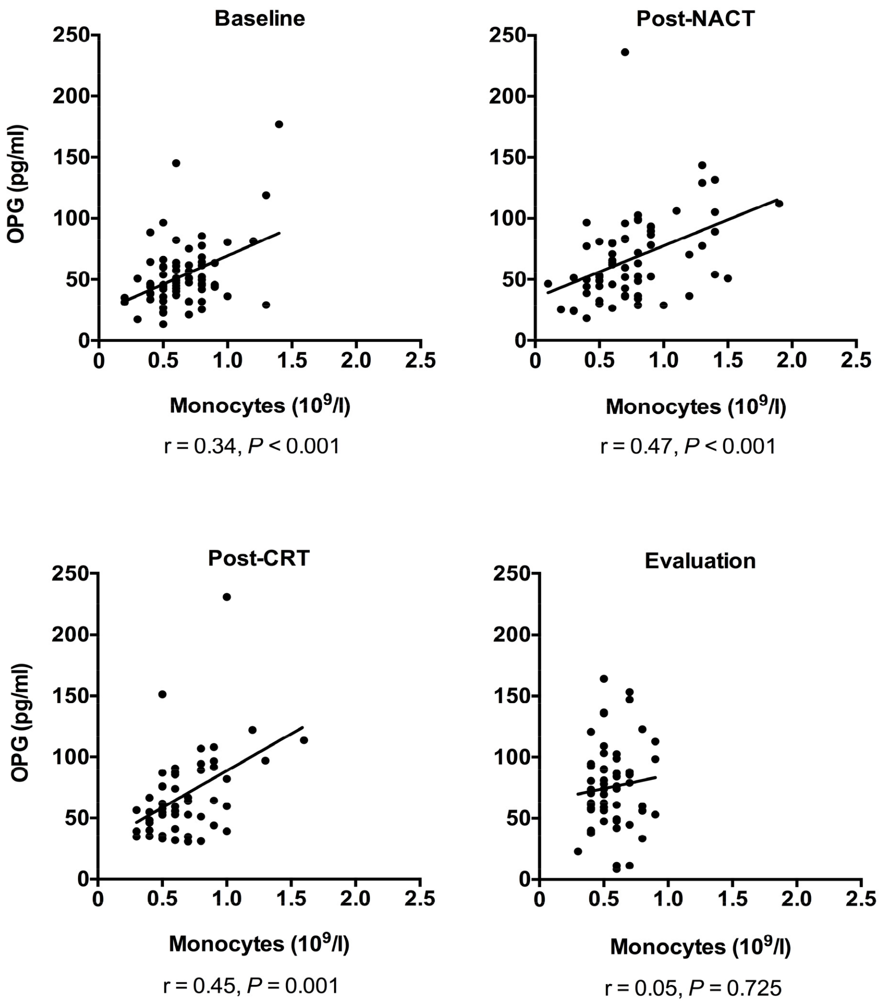

Supplementary Figure S1: Correlations between serum osteoprotegerin (OPG) levels and actual monocyte counts. Values of OPG (assessed by the single-parameter immunoassay) and monocytes at baseline (*n* = 71), following four weeks of induction neoadjuvant chemotherapy (post-NACT; *n* = 64), at completion of the sequential 5-week course of chemoradiotherapy (post-CRT; *n* = 50), and at evaluation of the neoadjuvant treatment four weeks later (*n* = 56) were compared by Pearson product correlation.

Supplementary Table S1: Progression-free survival – multivariate analysis

	HR (95% CI)	*P*-value
OPG (age-adjusted)	2.55 (0.99–6.52)	0.051
Hemoglobin	0.00 (0.00–0.51)	0.030
ESR	0.72 (0.30–1.69)	0.451

Adjusted hazard ratio (HR) with 95% confidence interval (CI) was calculated by Cox regression analysis. Abbreviations: OPG, osteoprotegerin; ESR, erythrocyte sedimentation rate.

Supplementary Table S2: Early alterations in serum osteoprotegerin – clinical parameters

		Total	Increase	No increase	*P*-value
		n (%)	*n* (%)	*n* (%)	
Sex	Female	25 (43)	17 (46)	8 (38)	
	Male	33 (57)	20 (54)	13 (62)	0.594
TN stage	T2–3	39 (67)	26 (70)	13 (62)	
	T4	19 (33)	11 (30)	8 (38)	0.569
	N0–1	13 (22)	11 (30)	2 (10)	
	N2	44 (78)	26 (70)	18 (90)	0.111
	ND	1		1	
ypTN stage	ypT0–2	33 (57)	23 (62)	10 (50)	
	ypT3–4	24 (43)	14 (38)	10 (50)	0.411
	ND	1		1	
	ypN0	40 (69)	26 (70)	14 (70)	
	ypN1–2	17 (31)	11 (30)	6 (30)	1.000
	ND	1		1	
TRG score	TRG 1–2	41 (71)	30 (81)	11 (55)	
	TRG 3–5	16 (29)	7 (19)	9 (45)	0.062
	ND	1		1	
CTCAE score	CTCAE 0–2	38 (66)	25 (68)	13 (62)	
	CTCAE 3	7 (12)	5 (14)	2 (10)	1.000
	ND	13 (22)	7 (19)	6 (28)	

Patients' changes in the serum level (assessed by single-parameter immunoassay) from baseline to completion of induction neoadjuvant chemotherapy were categorized into cases with (n = 37) or without (n = 21) increase. Distribution of the listed variables between the two categories was compared using Chi-square test or Fisher's exact test as appropriate. Abbreviations: CTCAE, Common Terminology Criteria for Adverse Events; TN, tumor-node; TRG, tumor regression grade; ypTN, histologic TN stage.
